# Helping International Medical Graduates Transition to the National Health Service: A Two-Cycle Quality Improvement Project (QIP)

**DOI:** 10.7759/cureus.82319

**Published:** 2025-04-15

**Authors:** Nishma B Patel, Kapilraj Ravendran, Kowthaman Balagumar, Ahsan Z Raja, Sonny Vaja

**Affiliations:** 1 General Medicine, George Eliot Hospital NHS Trust, Coventry, GBR; 2 Medicine, Gradscape, London, GBR; 3 Trauma and Orthopaedics, East and North Hertfordshire Teaching NHS Trust, Stevenage, GBR

**Keywords:** imposter syndrome, international medical graduates (imgs), nhs, quality improvement projects, uk imgs

## Abstract

International medical graduates (IMGs), when transitioning to the UK National Healthcare Service (NHS), typically get little constructive criticism for their work and have little opportunity to grow in their careers. Therefore, this quality improvement initiative's main goal was to increase the confidence of recently qualified physicians and IMGs as they transitioned to the NHS by using three systems: online instruction, in-person seminars, and an introduction guidebook. Our Quality Improvement Project (QIP) underwent two Plan, Do, Study, Act (PDSA) cycles to better IMGs' transition to the NHS, mimic a resident physician's life, comprehend the portfolio system, increase competency, and lessen imposter syndrome. Three different formats were used for each PDSA: induction manuals, online instruction, and in-person practical workshops. The surveys were filled out by 40 individuals in all, including nine UK national IMG students studying in Bulgaria and 31 recently graduated IMG physicians. The handbook showed improvement in confidence level, a mean increase of 1.56 (standard deviation (SD) 1.043, 95% confidence interval (CI) 0.238-2.173), p-value < 0.001, inperson workshop had a mean increase of 2.60 (SD 1, 95%CI 2.817-3.013, p-value < 0.001) and online sessions showed an increase across all teaching. This QIP brought to light the many difficulties experienced by non-UK-qualified doctors when they transitioned to practice in the UK. For IMGs, it is essential to implement an induction program in addition to ongoing instruction and mentorship.

## Introduction

Although they comprise 41% of the United Kingdom's medical profession, international medical graduates (IMGs) frequently hold solitary positions, receive little constructive feedback on their work, and encounter limited opportunities for career advancement, such as entry to training programmes [1]. More foreign doctors are entering the workforce than domestic physicians [2]. Eastern and central Europe are seeing an increase in the number of British physicians earning medical degrees. According to the British Medical Journal (BMJ), nearly 13,000 (4.3%) doctors on the General Medical Council (GMC)’s medical register have primary medical qualifications from eastern and central European countries; of these, 22% (2,910) are United Kingdom (UK) nationals [3]. IMGs then relocate to the UK to progress in their careers and benefit from the UK's esteemed medical education and training standards [4]. However, moving to a new country and adjusting to a different healthcare system is not a straightforward process. Career progression may be influenced by the social and educational challenges that accompany this transition [4]. When they first start working in the UK, most foreign medical graduates secure a non-training position, such as a trust grade role [4]. Since the trust employs them, trust-grade physicians are not subject to Royal College or deanery regulations [4]. Trust-grade physicians are expected to fulfil the competencies at their respective grade level and work alongside their trainee colleagues [4].

The National Health Service (NHS) employs 37% of doctors who were not trained in the UK, according to data from 2019. Eleven percent qualified from the European Economic Area (EEA), while 26% qualified from outside the UK/EEA [5]. Any doctor, irrespective of their background or ethnicity, may find it challenging to adjust to UK medical ethics and culture, as explained in the GMC Welcome to UK practice course [6]. In addition, the GMC's study on unequal achievement examined the additional risks faced by IMGs due to their lack of familiarity with UK examinations, success in college exams and recruitment processes, cultural disparities, poor peer connections, and system fit [6].

There is evidence that ethical decision-making in medical practice differs depending on the culture and jurisdiction, especially when it comes to topics like consent/information sharing, end-of-life decision-making, and the role of the family, which can lead to breakdown in communications [7-11].

The challenges and educational barriers that overseas medical graduates face in the UK are not well documented, and there is even less information available regarding the experiences of those who have held a trust-grade position. Consequently, the primary aim of this quality improvement initiative was to enhance the confidence of newly qualified doctors and IMGs in their transition to the NHS through three systems: an induction handbook, in-person workshops, and online teaching.

## Materials and methods

Gradscape is a non-profit organisation that aims to ease the transition from an IMG to working within the NHS by providing free advice and teaching to work as a UK foundation doctor.

The Quality Improvement Project (QIP) team included four doctors who have completed foundation training as trust-grade doctors, two foundation year 2 non-training trust-grade doctors, two locum IMG doctors, and three recent IMG graduates. Five doctors (N.B.P., K.R., K.B., A.Z.R., and S.V.) currently working were able to identify problem areas and ensure that the information covered in the sessions was accurate and appropriate.

In the majority of healthcare systems, quality improvement initiatives often utilise the Plan, Do, Study, Act (PDSA) cycles [12,13]. These cycles provide a framework for evaluating modifications iteratively to enhance the standard of current healthcare systems [12,13].

Two PDSA cycles were carried out on our QIP to enhance the transition of IMGs to the NHS, simulate the life of a resident doctor, understand the portfolio system, improve competency, and reduce imposter syndrome. Each PDSA was conducted across three categories: induction handbook, online teaching, and in-person practical workshops.

PDSA cycle 1

IMGs who were students or newly qualified IMG doctors were given a questionnaire (see Appendix A) and asked to answer questions on confidence using a Likert scale prior to receiving an induction handbook, online teaching, and attending an in-person workshop. They were also asked what they would like to know and what should be included to remove imposter syndrome and ease the transition.

PDSA cycle 2

Subsequently, online teaching, in-person workshops, and a handbook were developed as part of the implementation following data collection from PDSA cycle 2. Once again, IMG students and newly qualified IMG doctors were given a questionnaire (Appendix B) and asked to respond to their confidence using a Likert scale after the intervention.

One month after the three interventions, data were gathered using a questionnaire (Appendix C). A questionnaire (Appendix D) evaluated the doctor's confidence and level of satisfaction with each intervention. At the conclusion of the questionnaire, participants had the option to provide written descriptive comments. Following their unique experiences, they were invited to suggest improvements for each intervention.

Analysis of variance (ANOVA) and T-test were used to define the statistical significance of our results

The QIP project was registered at the Royal National Orthopaedic Hospital, with registration number RNOH QIP QI024.

## Results

A total of 40 participants completed the questionnaires - 31 newly graduated IMG doctors and nine UK national IMG students studying in Bulgaria.

Table 1 illustrates the confidence levels and improvements observed after each of the seventeen online lessons. The online teaching indicates an overall increase in confidence levels following each lesson, as displayed in Table 1.

**Table 1:** Confidence levels and improvements observed after each of the 17 online lessons The Likert scale was used with a scale of 1-5, 1 being not confident at all and 5 being extremely confident FY1: foundation year one, SD: standard deviation, CI: confidence interval, CV: curriculum vitae, CasTest: cardiac arrest scenario test, ep: episode, PE: pulmonary embolism, DVT: deep vein thrombosis, VTE: venous thromboembolism, MSE: mental state examination

TOPIC	Mean Before	Mean After	Mean difference	Mean Difference SD	Mean Difference CI	p-Value
DOCUMENTATIONS AND JOBS OF A FY1	3.31	4.69	1.375	1.204	0.733-2.017	<0.001
GUIDES TO AUDITS & QIPS	2.29	4.51	2.286	1.326	1.520-3.051	<0.001
Approaches to Difficult conversations with patients & family	2.75	4.38	1.625	1.188	0.632-2.618	<0.001
A to E protocols and handling emergencies as FY1	3.07	4.33	1.267	1.223	0.590-1.944	<0.001
Interview Technique and CV Prep	3.03	4.56	1.529	1.261	1.089-1.969	<0.001
Build a Portfolio as a FY1 and Appraisal	3.10	4.50	1.400	1.501	0.698-2.102	<0.001
Be the FY1 – Interactive Session	3.12	4.50	1.375	1.406	0.198-2.552	<0.001
ALS CasTest ep1	2.50	4.28	1.778	1.060	1.251-2.305	<0.001
ALS CASTest ep2	3.20	4.50	1.3	1.418	0.286- 2.314	<0.001
ALS Protocol	3.44	4.56	1.111	0.928	0.398-1.824	<0.001
Electrolyte Disturbances and Interpretation of Blood Gases	3.23	3.77	0.538	0.776	0.069-1.008	<0.001
E-horus and Completing Competencies	3.10	4.51	1.410	1.397	0.358-2.651	<0.001
Approaches to chest Pain	2.50	4.28	1.778	1.060	1.251-2.305	<0.001
PE, DVT and VTE Prophylaxis	2.75	4.38	1.625	1.188	0.632-2.618	<0.001
Approach to Geriatrics	2.29	4.51	2.286	1.326	1.520-3.051	<0.001
Assessing and Handling Intoxication	3.03	4.56	1.529	1.261	1.089-1.969	<0.001
Capacity Assessment, MSE and Sectioning	3.20	4.50	1.3	1.418	0.286- 2.314	<0.001

Table 2 also shows significant improvement following in-person workshops, which helped participants gain more confidence in practical skills. Table 2 demonstrates overall confidence for two workshops held in August 2025 and October 2025. The workshops involved assessing unwell patients, good documentation, physical examination, gaining consent, breaking bad news, analysing ECGs and imaging, catheterisation, cannulation, PR examination, suturing skills, and Advanced Trauma Life Support.

**Table 2:** Overall confidence for two in-person workshops held in August 2025 and October 2025 The Likert scale was used with a scale of 1-5, 1 being not confident at all and 5 being extremely confident. SD: standard deviation, CI: confidence interval

	Mean before	Mean after	Mean difference	Mean difference SD	Mean difference CI	p-value
Workshop 1	1.84	4.44	2.60	1	2.817-3.013	<0.001
Workshop 2	1.84	4.44	2.60	1	2.817-3.013	<0.001

Table 3 reveals that the participants gained increased confidence and insight into working as a foundation doctor in the UK. The handbook included guidance on producing quality documentation, writing discharge summaries, understanding ward rounds, proficient A to E assessments, handling common emergencies, the primary responsibilities of a foundation doctor, key drug prescriptions, ordering scans, managing on-call duties, effective SBAR communication, medical terminology, fluid prescription, crafting an impressive CV, interview techniques, breaking bad news, engaging with complex patients, assessing capacity, and a guide to conducting QIPs and audits. Table 3 illustrates our findings on the improvements following our induction booklet.

**Table 3:** Findings on the improvements following our induction booklet The Likert scale was used with a scale of 1-5, 1 being not confident at all and 5 being extremely confident. SD: standard deviation, CI: confidence interval

	Mean before	Mean after	Mean difference	Mean difference SD	Mean difference CI	p-value
Induction hand booklet	2.97	4.53	1.56	1.043	0.238 -2.173	<0.001

The participants' written descriptive comments praised the three treatments for boosting their self-confidence and readiness to practice medicine in the UK. However, they noted that many of the lessons were conducted on artificial mannequins, and they were not afforded the opportunity to practice on real patients. They also remarked that several of the seminars were lengthy and content-heavy.

Each of the teachings was also Continuing Professional Development (CPD) approved by the Royal College of Surgeons, Edinburgh, which benefited participants in their continuous learning and professional development while seeking jobs.

## Discussion

IMGs working in trust-grade positions face several obstacles, including educational difficulties [4]. One of the primary barriers was the absence of a formal induction programme. Despite the significance of the topics covered in the introduction programme, many individuals felt it was too much information to absorb in two days and lacked relevance [4]. Extending the introduction period and incorporating more medically pertinent subjects, such as the supervisor's role, the value of evaluation and revalidation, the types of career portfolios required, and medicolegal and ethical considerations, could improve this [4]. A comprehensive understanding of these subjects would assist physicians in feeling more oriented, potentially enhancing their self-esteem and confidence [4]. Our findings showed that a structured approach as to what topics would benefit IMGs will help improve induction, remove imposter syndrome, and build confidence.

Given that employers are twice as likely to send overseas medical graduates to the GMC as to UK graduates, information on the necessity of medicolegal coverage is particularly crucial [4]. Furthermore, whereas 95% of UK graduates are aware of the need for medicolegal coverage before working in the NHS, only about a quarter (26.5%) of overseas medical graduates are informed about this requirement, according to research by Jalal et al. [12]. This could be used for further scope for further studies, as this was absent from our QIP.

The absence of a study budget not only leads to increased expenses and fewer educational opportunities but also diminishes participants’ confidence, as they feel disadvantaged compared to their peers enrolled in training programmes [4]. IMGs are also concerned about the unequal distribution of study leave and taster days allowed for trust-grade physicians [4]. Addressing these issues is essential to ensure that all physicians receive the same treatment [4]. This would foster a sense of acceptance or belonging, enhance their self-esteem and motivation, and positively impact the systems in which they operate [4]. Introducing workshop-style teaching showed this can help build confidence for IMGs, which in turn can lead to better prospects.

IMGs believe that they face challenges with various aspects of communication despite the majority having completed their core medical qualifications in English [4]. Transitioning to a new healthcare system is often tricky. A study by Wong and Lohfeld (2007) outlines the evolution of an IMG's experience from feeling like an outsider to eventually settling in, describing it in three phases: 'loss, through disorientation, to adaptation'. This transition period can be expedited and made smoother through specific actions [14]. Upon commencing work in the UK, non-UK-trained physicians frequently encounter communication difficulties. These may range from limited proficiency in English to more subtle misinterpretations of nonverbal cues and social and behavioural norms [5]. All physicians must adapt to and engage with diverse communication styles due to the multicultural nature of UK society, which can be incredibly challenging for those trained abroad [5]. Our communication skills workshop showed that IMGs are more confident when given a whistle stop and practice for communication skills needed to be an NHS doctor.

A survey of medical graduates in the UK found that upon commencing foundation postings, they lacked comprehension in non-clinical areas, such as ethics and law [5]. Non-UK graduates are likely to have similar requirements, particularly if UK graduates need in-practice training and support for ethical decision-making at the onset of their clinical practice [5]. Our QIP showed that specific targeted workshops will help IMGs with foundation competencies.

Ajaz et al.'s study showed that on a scale of 1-5, 69% of respondents gave their initial NHS induction a rating of 3 or lower. When they first started, 44% of the physicians did not get a separate departmental induction. A separate IMG-specific introduction would have been beneficial, according to 92% of respondents [15].

In order to improve the performance and retention of foreign physicians, healthcare professionals and organizations must be aware of the clinical and cultural disparities [16]. Mentors and supervisors also gain from this information as it enables them to comprehend cultural differences and assist foreign physicians in integrating into the NHS, which has its own unique culture [16]. Mentorship throughout the workshops helped IMGs understand their disparities and gave a clear understanding of which aspects need improving.

In addition to the current inductions in local hospitals, an obligatory nationwide induction program for foreign physicians will be beneficial [17]. Sessions on 'culture', especially the NHS and the British way of life, as well as clinical skills, might be incorporated into the curriculum [17]. Content should concentrate on the unique requirements of IMGs, with particular attention to medicolegal and ethical problems, as well as the NHS's principles and ethos, in order to increase the effectiveness of inductions [18]. In addition, the induction programs have to run for more than a day - ideally, a whole week [18].

One crucial intervention that might be used to facilitate IMGs' seamless integration into the NHS system is clinical shadowing or a period of clinical attachment [19]. The NHS has long employed shadowing as a new physician induction technique, especially for recently trained physicians joining the UK foundation program, with positive results [19]. According to similar research, shadowing for extended periods of time has greater advantages than shadowing for just one week [20].

Furthermore, it appears that IMGs view their move into the NHS as an ongoing process [21]. Enhancing their mentorship via more regular contacts with their supervisors and increasing NHS staff understanding of IMG requirements are also necessary, even if shadowing and introduction provide support at the start of the transfer phase [21].

This QIP shows the importance of a compulsory induction programme and regular teaching for IMGs to ease the transition, reduce imposter syndrome, and improve confidence levels.

Senior doctors are required to coach trainees, and training postings offer a supportive learning environment. Senior coworkers who provided guidance in morally challenging circumstances were commended by training course participants. On the other hand, non-training positions are frequently temporary, lack a designated line manager, and assume the doctor can manage independently. The isolation that many people feel in an unsupported clinical setting is exacerbated by the absence of an established peer network for non-UK-qualified physicians, especially in the early years of employment. The issue of assistance in these positions is urgent, given the high number of non-UK-qualified physicians in non-training positions and the decline in training post chances for these physicians.

Limitations

Certain restrictions exist on this quality improvement endeavour. First, the extremely small sample size diminishes the validity and, thus, generalizability of our findings. Lastly, the lack of a control group in our study makes it more difficult to link our results to the therapies used.

## Conclusions

Through this study, the author identified the difficulties encountered by foreign medical graduates in trust-grade positions and, where feasible, proposed solutions. During their transition to practice in the UK, non-UK-qualified physicians faced numerous challenges, which this QIP highlighted. Implementing an induction programme alongside regular teaching and mentorship for IMGs is crucial. Our QIP illustrates that an effective induction booklet and tailored teaching to address IMG needs can facilitate a smoother transition.

## Appendices

Appendix A

Introducing Gradscape CPD-Approved NHS Essentials: Navigating Your Medical Career - An In-Person Two-Day Workshop Sponsored by the MDU 

The Gradscape team extend their sincerest gratitude to you for attending our workshop sponsored by MDU. This is CPD-approved by the Royal College of Surgeons of Edinburgh. Please note that you will be awarded CPD points for the successful completion of this form.

* Indicates required question

1.

Name *

2.

Email Address *

3.

Place of Study *

4.

Year Of Study *

Mark only one oval.

1st Year

2nd Year

3rd Year

4th Year

5th Year

6th Year

Other

5.

Do you think you learned from this workshop? *

Mark only one oval.

Yes

No

6.

On a scale of 1-5, with 1 being very little, to 5 being a lot, how much do you feel you learned from our two day workshop? *

Mark only one oval.

1 - Very little

2

3

4

5 - A lot

7.

On a scale of 1-5, with 1 being not confident, to 5 being very confident, how confident were you in your knowledge prior to attending this course? *

Mark only one oval.

1 - Not confident

2

3

4

5 - Very confident

8.

On a scale of 1-5, with 1 being not confident, to 5 being very confident, how confident are you after this workshop? *

Mark only one oval.

1 - Not confident

2

3

4

5 - Very confident

9.

In your own words, what did you learn from this event? *

10.

Please describe 3 aspects of the event you enjoyed. *

11.

Please describe 3 aspects of the event that could be improved. *

12.

On a scale of 1-5, with 1 being poor, to 5 being very excellent how would you rate the talk on GMC registration?

Mark only one oval.

1

2

3

4

5

13.

On a scale of 1-5, with 1 being poor, to 5 being very excellent how would you rate the talk on OETS/IELTS *

Mark only one oval.

1

2

3

4

5

14.

On a scale of 1-5, with 1 being poor, to 5 being very excellent how would you rate the talk on CV and Interview Techniques *

Mark only one oval.

1

2

3

4

5

15.

On a scale of 1-5, with 1 being poor, to 5 being very excellent how would you rate the talk on Introduction in conducting a research *

Mark only one oval.

1

2

3

4

5

16.

On a scale of 1-5, with 1 being poor, to 5 being very excellent how would you rate the talk on conducting a QIP/Research *

Mark only one oval.

1

2

3

4

5

17.

On a scale of 1-5, with 1 being poor, to 5 being very excellent how would you rate the talk on Portfolio *

Mark only one oval.

1

2

3

4

5

18.

On a scale of 1-5, with 1 being poor, to 5 being very excellent how would you rate the workshop on  NG tube venepuncture and cannulation *

Mark only one oval.

1

2

3

4

5

19.

On a scale of 1-5, with 1 being poor, to 5 being very excellent how would you rate the workshop on catherisation and examining a patient *

Mark only one oval.

1

2

3

4

5

20.

On a scale of 1-5, with 1 being poor, to 5 being very excellent how would you rate the workshop on introduction to ALS Castest *

Mark only one oval.

1

2

3

4

5

21.

On a scale of 1-5, with 1 being poor, to 5 being very excellent how would you rate the workshop on assessing an unwell patient (A to E) *

Mark only one oval.

1

2

3

4

5

22.

On a scale of 1-5, with 1 being poor, to 5 being very excellent how would you rate the workshop on suturing *

Mark only one oval.

1

2

3

4

5

23.

On a scale of 1-5, with 1 being poor, to 5 being very excellent how would you rate the workshop on physical examination and clerking *

Mark only one oval.

1

2

3

4

5

24.

If possible, what changes would you have made to the event? This can include any inclusions, removals or modifications. *

25.

Did the event meet your expectations? *

Mark only one oval.

Yes

No

Other:

26.

Do you have any other questions to ask after this event? *

27.

On a scale of 1-5, with 1 being poor, to 5 being excellent, how would you rate this event? *

Mark only one oval.

1 - Poor

2

3

4

5 - Excellent

28.

Overall, did the event meet your expectations? *

Mark only one oval.

Yes

No

29.

Would you recommend this course to a friend? *

Mark only one oval.

Yes

No

30.

Where did you hear about our course from? *

Check all that apply.

Word of mouth

Friends/Family

Social media

Other:

31.

Finally, do you have any other feedback to provide on any aspect of this event?

Appendix B

QIP on Teaching series - Is it helpful for IMGs transitioning to the NHS 

The Gradscape team extend their sincerest gratitude to you for kindly filling in this form

* Indicates required question

1.

Name *

2.

Email Address *

3.

Place of Study/Work *

4.

Year Of Study *

Mark only one oval.

1st Year

2nd Year

3rd Year

4th Year

5th Year

6th Year

Other

5.

How prepared and confident are you to work in the NHS? *

Mark only one oval.

Not very confident

1

2

3

4

5

Very Confident

6.

How much information do you think you have in preparing to work for the NHS *

Mark only one oval.

Not a lot

1

2

3

4

5

A lot

7.

In terms of job specification as FY1/FY2 - Are you aware of what is required of a FY1/FY2 *

Mark only one oval.

Not much

1

2

3

4

5

Very much

8.

How confident are you with your CV in preparing to apply for job? *

Mark only one oval.

Not confident

1

2

3

4

5

Very Confident

9.

How confident are you of documentation and jobs a Foundation Doctor is required to do? *

Mark only one oval.

Not very confident

1

2

3

4

5

Very confident

10.

How confident are you are regarding having difficult conversations as a foundation doctor *

Mark only one oval.

Not confident

1

2

3

4

5

Very confident

11.

How confident are you are managing emergencies required of a foundation doctor *

Mark only one oval.

Not confident

1

2

3

4

5

Very confident

12.

What teaching/webinars would you find useful *

13.

Would a handbook be useful *

Mark only one oval.

Yes

No

Other:

14.

What would you like the handbook (pdf) to contain? *

15.

Finally, do you have any other feedback to provide on any aspect of today's talk?

Appendix C

QIP on Teaching series - Is it helpful for IMGs transitioning to the NHS 

The Gradscape team extend their sincerest gratitude to you for kindly filling in this form

* Indicates required question

1.

Name *

2.

Email Address *

3.

Place of Study/Work *

4.

Year Of Study *

Mark only one oval.

1st Year

2nd Year

3rd Year

4th Year

5th Year

6th Year

Other

5.

How prepared and confident are you to work in the NHS after receiving the handbook? *

Mark only one oval.

Not very confident

1

2

3

4

5

Very Confident

6.

How much information do you think you have in preparing to work for the NHS after receiving handbook *

Mark only one oval.

Not a lot

1

2

3

4

5

A lot

7.

In terms of job specification as FY1/FY2 - Are you aware of what is required of a FY1/FY2 *

Mark only one oval.

Not much

1

2

3

4

5

Very much

8.

How confident are you with your CV in preparing to apply for job? *

Mark only one oval.

Not confident

1

2

3

4

5

Very Confident

9.

How confident are you of documentation and jobs a Foundation Doctor is required to do? *

Mark only one oval.

Not very confident

1

2

3

4

5

Very confident

10.

How confident are you are regarding having difficult conversations as a foundation doctor *

Mark only one oval.

Not confident

1

2

3

4

5

Very confident

11.

How confident are you are managing emergencies required of a foundation doctor *

Mark only one oval.

Not confident

1

2

3

4

5

Very confident

12.

What teaching/webinars di you find useful *

13.

Was the handbook be useful *

Mark only one oval.

Yes

No

Other:

14.

Finally, do you have any other feedback to provide on any aspect of today's talk?

Appendix D

Feedback form for CPD Teaching Series sponsored by the MDU -  Blueprints IMG CPD approved talk sponsored by the MDU - Be the FY1 (An interactive session) - Kapilraj Ravendran - 28th April 2024 

The Gradscape team extend their sincerest gratitude to you for attending our talk. We kindly request for you to fill in a short feedback form on the talk you just attended. Please note you will be awarded CPD points for successful completion of this form.

* Indicates required question

1.

Name *

2.

Email Address *

3.

Place of Study/Work *

4.

Year Of Study *

Mark only one oval.

1st Year

2nd Year

3rd Year

4th Year

5th Year

6th Year

Other

5.

Do you think you learned from today's talk? *

Mark only one oval.

Yes

No

6.

On a scale of 1-5, with 1 being very little, to 5 being a lot, how much do you feel you learned from today's talk? *

Mark only one oval.

1 - Very little

2

3

4

5 - A lot

7.

On a scale of 1-5, with 1 being not confident, to 5 being very confident, how confident were you in your knowledge of today's talk topic prior to attending this course? *

Mark only one oval.

1 - Not confident

2

3

4

5 - Very confident

8.

On a scale of 1-5, with 1 being not confident, to 5 being very confident, how confident are you on the talk topic after the presentation? *

Mark only one oval.

1 - Not confident

2

3

4

5 - Very confident

9.

In your own words, what did you learn from this event? *

10.

Please describe 3 aspects of the talk you enjoyed. *

11.

Please describe 3 aspects of the talk that could be improved. *

12.

If possible, what changes would you have made to the talk? This can include any inclusions, removals or modifications. *

13.

Did the speaker meet your expectations in delivering the talk? *

Mark only one oval.

Yes

No

Other:

14.

Do you have any other questions to ask the speaker, or regarding the talk? *

15.

On a scale of 1-5, with 1 being poor, to 5 being excellent, how would you rate today's speaker? *

Mark only one oval.

1 - Poor

2

3

4

5 - Excellent

16.

Overall, did the talk today meet your expectations? *

Mark only one oval.

Yes

No

17.

Would you recommend this course to a friend? *

Mark only one oval.

Yes

No

18.

Where did you hear about our course from? *

Check all that apply.

Word of mouth

Friends/Family

Social media

Other:

19.

Finally, do you have any other feedback to provide on any aspect of today's talk?
